# Literacy and motivation for the prevention and control of hypertension among female community health volunteers: a qualitative study from Nepal

**DOI:** 10.3402/gha.v8.28254

**Published:** 2015-12-15

**Authors:** Dinesh Neupane, Craig S. Mclachlan, Rupesh Gautam, Shiva Raj Mishra, Michael Thorlund, Mette Schlütter, Per Kallestrup

**Affiliations:** 1 Center for Global Health, Department of Public Health, Aarhus University, Aarhus, Denmark; 2 Rural Clinical School, University of New South Wales, Sydney, Australia; 3 Unit for Health Promotion Research, University of Southern Denmark, Esbjerg, Denmark; 4 Nepal Development Society, Chitwan, Nepal; 5 Faculty of Social Sciences, Aalborg University, Aalborg, Denmark; 6 Department of Culture and Society, Aarhus University, Aarhus, Denmark

**Keywords:** community health workers, female community health volunteer, focus group discussion, hypertension, prevention, primary care, health literacy, Nepal

## Abstract

**Background:**

The prevalence of hypertension is increasing in Nepal. Thus, there is a need for a programme to improve primary healthcare. One possibility is to assign prevention, diagnosis, and treatment of hypertension to female community health volunteers (FCHVs).

**Objective:**

To assess literacy and motivation to be involved in a hypertension prevention and control programme in Nepal among FCHVs.

**Design:**

Five focus group discussions (FGDs) were conducted with a total of 69 FCHVs in Lekhnath municipality, Kaski district, Nepal. Seven themes were developed on the basis of data collection: 1) knowledge about hypertension; 2) risk factors of hypertension; 3) prevention and control of hypertension; 4) access to treatment for hypertension in the community; 5) learning about blood pressure measurement; 6) ability to raise blood pressure awareness in the community; 7) possible challenges for their future involvement. Data were analysed using the thematic analysis approach.

**Results:**

FCHVs have some knowledge about diagnosis, risk factors, and complications of hypertension. General unanimity was observed in the understanding that hypertension and risk factors needed to be addressed. The willingness of FCHVs to contribute to prevention, control, and management was strong, and they were confident that with some basic training they could obtain skills in hypertension management.

**Conclusions:**

Despite limited knowledge about hypertension, FCHVs expressed willingness and readiness to be trained in hypertension management. This study supports the possibility of involving FCHVs in prevention and control of hypertension in Nepal.

Hypertension plays a significant role in heart disease, stroke, kidney failure, disability, and premature death (1). Hypertension alone accounts for 9.4 million cardiovascular deaths annually (2), and it is the leading risk factor for global burden of disease (GBD) and mortality (3). There is a high prevalence of hypertension (over 30%) in the population of Nepal (4); prevalence has increased three-fold over the last three decades (5), suggesting an urgent need for developing a community programme. One possible solution is to shift prevention, diagnosis, and treatment of hypertension and other non-communicable diseases (NCDs) to female community health volunteers (FCHVs), a type of community health workers (CHWs) in Nepal (4). In Nepal, there are over 50,000 FCHVs working to improve the health of their communities (6), and they are highly recognised for the contribution they have made to improve maternal and child health (7). Nepal's Ministry of Health and Population relies on the FCHVs to act as health resources in their villages, educate their neighbours about important health issues, and provide basic health services in the absence of other healthcare resources. FCHVs are selected by members of Mothers’ Groups for Health with the help of local health facility staff. FCHVs receive 18 days of basic training in two steps (9 + 9 days), covering selected primary healthcare components. The current role of the FCHVs is to promote safe motherhood, child health, and family planning (6). According to a national FCHV survey, the median age of FCHVs is 37 years and 42% have completed primary school or have a higher educational level (8).

Despite FCHVs’ positive record of collaborating with the official health system, they have yet to be mobilised in the prevention, control, and treatment of hypertension. The FCHVs serve as a unique link between the health facilities and local communities, and they have the potential to play an important role in promoting knowledge and awareness for healthier lifestyles and thereby reduce the risk of hypertension. FCHVs are the Nepali equivalent of community health workers elsewhere (9). Based on the experience of other similar low and middle income countries, it can be expected that these health cadres can also play a role in addressing health problems not usually considered their domain (10–14). In Nepal, FCHVs have demonstrated that they are capable of diagnosing problems like acute respiratory infection (ARI) among children using an ARI timer, and were also entrusted with providing treatment with antibiotics. Using FCHVs for this responsibility at that time was controversial, as most of the FCHVs were illiterate (15). But later, they were entrusted with a programme addressing the community-based management of childhood illness in which they demonstrated their capacity despite the limitation of illiteracy (16).

In an effort to design and implement an effective programme for hypertension, it is important to investigate and include the FCHVs’ knowledge and beliefs about hypertension. Our study aims to explore literacy and motivation among FCHVs and their potential role in the management of hypertension at community level. The main aim of the analysis was to identify important factors when developing an intervention programme for community-based management of hypertension in Nepal.

## Methods

### Study setting

FCHVs who work in Lekhnath municipality, Kaski district, Nepal, were asked to participate in focus group discussions (FGDs). The municipality was composed of 15 wards with a total population of 58,816. Having approximately one FCHV for a population of 500, the municipality in addition has one primary healthcare centre, two urban health centres, and three sub-health posts.

### Procedure

An FGD gathers people from similar backgrounds or experiences to discuss a specific topic of interest to the researcher (10). FGDs are useful in exploring the findings of surveys that cannot be explained statistically, in exploring the opinions and views of the participants who share common experiences and also to learn the locally used terms relevant to the topic investigated, which cannot be derived from closed-ended quantitative surveys (11). This method of data collection was appropriate as it aimed to fulfil all the above-mentioned objectives. It complemented the findings of a quantitative survey and gave the freedom of expression of opinions to the FCHVs in a homogeneous setting.

FGDs took place at five different locations in the municipality. The first author moderated the FGDs. Five focus group sessions were conducted in different places, involving a total of 69 FCHVs. The selection of FCHVs aimed at covering all geographical areas, ethnicities, age groups, educational status, and years of experience as an FCHV. The median age of FCHVs was 42 years, ranging from 20 to 70 years. Ethnically, 2 were Dalit, 4 were disadvantaged Janajati, 7 were relatively advantaged Janajati, and 56 were upper caste. Of all the FCHVs, 57 were able to read and write without any problems. The mean year of experience as an FCHV was 14.5 years ranging from 4 months to 31 years.

Each FGD generally lasted between 60 and 90 minutes and was conducted in Nepali language. The FGD guide was adapted and modified from a similar study performed in South Africa (12). Participants in the FGDs were asked to discuss their understanding of hypertension with a particular focus on risk factors, prevention, and the situation in their community. Further details were considered including access to treatment, cultural practices, government programmes, future interest in measuring blood pressure, and providing health promotion messages. The first author identified when the discussions had reached saturation and made the decision to either continue or end the discussion.

### Data analysis

The FGDs were digitally recorded and later transcribed into English by one of the co-authors – a native Nepali speaker. The translation was again checked for correctness by the first author, who is also a native Nepali speaker. In addition, extensive notes were taken during the FGDs, and these were subsequently added when the recordings were reviewed and transcribed. A set of codes was developed and data were sorted and analysed thematically. Seven themes were developed: 1) knowledge about hypertension; 2) risk factors of hypertension; 3) prevention and control of hypertension; 4) access to treatment for hypertension in the community; 5) learning about blood pressure measurement; 6) ability to raise awareness in the community; and 7) possible challenges for future involvement.

### Ethical considerations

Ethical approval was obtained from the Ethics Committee of Nepal Health Research Council. Informed written consent was obtained from all participants before the FGDs took place. All participants were also informed that the FGDs were being recorded digitally and it was ensured that voice recordings would only be used by the research team and would be deleted from all digital archives after the study was completed.

## Results

### Theme 1: knowledge about hypertension

Most FCHVs referred to hypertension as high blood pressure (HBP) – ‘uchcha raktachaap’ in local language – and mentioned it alongside low blood pressure (LBP), which they considered an important community health problem, too. FCHVs mentioned that HBP was more common and more serious in the community. They also had some familiarity with the way hypertension is defined or measured. Some were more precise about the measurement than others, but most of the FCHVs had an idea about how to measure it. One FCHV said:

Although I hear a lot about the measurement of hypertension using terms like 120/80, 120/70 and so on, I do not understand what exactly it refers to.

Some, however, were unsure about the differences between HBP and LBP. One FCHV said:

I know that having a blood pressure at the level of 120/80 is normal. Higher than that is high blood pressure and lower than that is low blood pressure.

The FCHVs reported that hypertension manifests itself in the form of headache and dizziness, lethargy, and overall tiredness even when not doing any physically demanding work. One FCHV described the symptoms of hypertension as:

Headache, pain in the nerves, feeling irritated at something told by somebody that you don't approve of, feeling dizzy while moving around, feeling tired, tingling in head and having an uncomfortable feeling even while simply lying in bed are some of the symptoms of hypertension.

They also compared these symptoms with those of LBP, which they said manifested almost in the same way as HBP.

Low blood pressure has also been found to have some effects on the patients, with the expression of symptoms like headache, dizziness, drowsiness, and weak feelings in the limbs.

Almost all FCHVs suggested that hypertension was associated with paralysis. Participants made references to cases where people were suffering from disabilities following an attack (stroke). They also mentioned heart attack, stroke/taalu futnu, and sudden death. One FCHV said:

I have heard of instances from others where someone fell down to the ground all of a sudden or someone passed away suddenly while they were in the lavatory. When we inquired about the reason behind it, people told us that it was due to high blood pressure, which hit the heart and resulted in those unfortunate events. Some such people have survived but are now living with paralysis.

Many of the FCHVs believed that hypertension drugs were to be consumed on a regular basis for the rest of life and a failure to comply would put the patients at high risk of paralysis.

### Theme 2: risk factors of hypertension

Stress was mentioned most frequently by the FCHVs as a risk factor for hypertension. FCHVs revealed that they had observed stressful lifestyle and/or event/s could lead to hypertension. Lack of physical exercise was another risk factor pointed out as responsible for causing hypertension. However, it was also mentioned that being physically active alone might not be sufficient to prevent hypertension. Poor dietary habits were also a risk factor mentioned frequently by the FCHVs. However, others who were more specific about what kinds of food habits were considered risk factors for the disease believed that (excessive) consumption of oily food was responsible for developing hypertension. There were some other risk factors which revolved around what people decided to consume such as meat; excessive salty, sour and hot foods; vegetables and foods produced with excessive use of pesticides; and poultry fed with chemical feed. The change in people's dietary habit, specifically the shift from consuming homegrown foods and vegetables to commercial foods, was considered responsible for the current surge in the occurrence of hypertension. One FCHV said:

In the past, people used to consume homegrown foods and vegetables, and they also kept livestock for milk consumption. Many of them do not do it anymore.

FCHVs attributed increase in consumption of broiler chicken, which has more fat, as a factor of increasing prevalence of hypertension in their community. One said:

In the earlier days, we used to keep local chicken for meat consumption. Now we cannot buy local chicken anywhere, all we get is broiler chicken. I don't think it is a healthy practice.

However, it was also mentioned that following proper dietary practices or doing enough physical activities alone was not enough to prevent hypertension. They mentioned even the ones who are very physically active might have hypertension. One said:

It has been observed that even the ones who were very cautious about their food habits were found dead or paralyzed in bed in the morning.

Heredity was also believed to be a cause of hypertension. Increase in the amount of body fat and cholesterol were other isolated risk factors mentioned. Other reasons mentioned were age and comorbidities. Poor hygiene, rise in blood temperature, and the blood becoming ‘dirty’ were also mentioned as responsible for developing hypertension. Notably, most FCHVs seemed to be aware that there was more than one isolated risk factor responsible for the occurrence of hypertension; moreover, that avoidance of one risk factor might not necessarily prevent the disease altogether. This suggests their awareness of the multi-factorial causation of the condition.

### Theme 3: prevention and control of hypertension

The FCHVs seemed to make a distinction between ways to prevent and control hypertension although some were overlapping. Cutting down on oily foods, reducing salt consumption, regular monitoring of blood pressure, and avoidance of meat (some respondents were more detailed and specified red meat) were suggested as ways to prevent and control hypertension.

Others said that regular monitoring of blood pressure helps people stay informed and thereby to prevent hypertension. A healthy diet in general and/or controlling dietary habits to prevent hypertension was mentioned by others. One said:

If one loves their body and wants to keep it in good shape, they need to have a proper and healthy dietary practice.

Avoidance of alcohol, stress, vegetables sprayed with chemical pesticides, and the use of motorbikes for travelling short distances as well as controlling body weight and being physically active were other ways pointed to prevention of hypertension. Some were still unaware of ways to prevent the condition and were looking forward to learning about it.

Taking anti-hypertensive drugs regularly and consistently was pointed out as one way of controlling hypertension. Most FCHVs seemed to understand the importance of lifelong consumption of hypertension drugs and they also seemed to relate discontinuation or poor medication compliance to stroke and paralysis. One said:

If you have to take the medication for hypertension, you should not take it as a big deal and should just consider it an addition of one more thing to your daily routine of food consumption.

Some FCHVs had heard about and also seen some people practise walking barefooted in the grass with morning dew. Some of them even believed that if practised properly, this alone could help control the blood pressure. Reference was also made to an instance where someone practicing the walk without taking any medication to control his or her hypertension had collapsed. Maintaining a healthy diet; daily exercise; avoiding spicy, sour, or meat-based foods and alcohol; monitoring blood pressure regularly as well as increasing water consumption were also suggested as ways to prevent HBP.

Some FCHVs also believed that people with hypertension should not be abruptly told anything that could lead to a sudden surge of emotions. They believed this might even lead to an immediate death. FCHVs mentioned that adopting self-control in personal behaviour was necessary for preventing hypertension.

Adoption of home remedies such as consuming bitter foods, bitter gourd, and herbs (e.g. *Burjo*) was considered helpful in curbing HBP status and was pointed out as being practised in the communities.

In one rather peculiar case, it was mentioned that colostrums of cow and water buffaloes (which are considered delicacies among many Nepalese) should be avoided; otherwise hypertension would be aggravated. A case was mentioned where a person with hypertension had felt dizzy and started sweating profusely after consuming colostrums.

Continuing to take medications without monitoring was also implied as a wrong approach in one instance.

It is not a good idea to keep buying the same medication with the use of old prescription without consulting a doctor because the blood pressure keeps fluctuating and you don't know if it is high or low at a point if you don't keep it monitored frequently.

Knowledge on hypertension and the associated factors was also mentioned as a way to keep hypertension under control. There was a wish by participants to learn about therapeutic interventions and subsequently to practise proper blood pressure management.

We need to hear some words from the authority on how to control it. As of now, as FCHVs we do not have a clear understanding of how to control hypertension. But once instructed clearly on what to do, we do have the capacity to deliver in terms of controlling the disease.

### Theme 4: access to treatment for hypertension in the community

The FCHVs not only saw the lack of access to proper treatment of hypertension in their communities but also the lack of possibilities for assessing and monitoring blood pressure to prevent cardiovascular disease. Many FCHVs suggested it was not possible in their communities to have their blood pressure measured frequently. Thus, even when a person shows signs of hypertension, it is not possible for the FCHVs to monitor the blood pressure in the local setting. One FCHV said:

At present there are many elderly citizens who have to go to different places just to get their blood pressure examined. If we can provide them with this service, in their own local community, it will be a great help for them, too. We can even provide them the service at home.

It is further expressed that many people from uneducated families do not understand the consequences of hypertension and therefore do not bother to travel to a healthcare facility if they have symptoms of cardiovascular disease related to hypertension. This stresses the need for better community literacy concerning signs, symptoms, and consequences of hypertension. One FCHV said:

The people from educated families take the initiative to take those members of family who show some signs and symptoms of cardiovascular disease related to hypertension to the health care facilities, but those from uneducated families do not really bother.

They also mentioned that poor people face logistical difficulties. One FCHV added:

First of all, transportation is a major problem. There is no frequent bus service.

When hypertension has been detected, it is still difficult for people to get access to treatment. As there is no local facility offering treatment, people often have to travel far to consult a doctor. One FCHV expressed her opinion as:

If someone is not able to go to hospital in the city, there is no health facility available at the local community level. Those who can afford can have best health services, but it is not easy for people who cannot afford the costly treatment.

### Theme 5: learning about blood pressure measurement

The FCHVs in general were confident enough about their capacity to measure blood pressure after receiving training on using a manual sphygmomanometer. One said:

We can do it and it is not a big deal. It is just like looking at your watch. Once we know that a point in the reader says that certain point indicates high pressure and a certain point indicates that it is low pressure, it is just a matter of getting ourselves used to it.

But despite this confidence, there were also a modest realisation about one's capacity.

We are grass root level health volunteers and cannot claim to be able to achieve a high level expertise after one training session. We have been helping women and children through our activities at present and we are confident that we can also serve others at the individual capacity if we get a chance to learn about hypertension.

In addition to this modesty, there was an overall enthusiasm about the capacity to handle the responsibility and FCHVs believed that they have demonstrated similar abilities in the past. They cited the successful role they had played in correctly diagnosing pneumonia among children (0–59 months) by using a one-minute ARI timer to count the respiratory rate and then either treat the children or refer them to a health facility depending on disease severity; they said that this was previously believed to be too complicated with their level of training.

People found it hard to believe that we could manage pneumonia in children, but now we have been able to identify pneumonia in young children after the training. Even though the timer was a new tool to us we mastered using it. I think that measuring blood pressure is not any different; we will try our best to learn it and master it.

The optimism and confidence exhibited by one FCHV sums up their willingness to undertake the responsibility. One said:

If people just like us can fly airplanes, why can't we do a task as simple as measuring someone's blood pressure?

### Theme 6: ability to raise blood pressure awareness in the community

FCHVs showed high levels of readiness to take on the task of spreading awareness about hypertension. Although they are not assigned directly to perform this task at present, they are involved in making people aware of some of the risk factors, for example, smoking. They believed that they could use the pre-existing platform like mothers’ group meetings for that purpose. One FCHV said:

We could, for example, during the mothers’ group meetings educate the women about hypertension in general, how to avoid it, how to tackle it with regular consumption of medication, to get blood pressure monitored at regular intervals, taking advice from the doctor on whether to take the medication or to manage the problem on their own.

The altruistic feeling was evident in their willingness to be a part of raising awareness concerning blood pressure.

Even if it means adding extra responsibility for no extra payment, we will be satisfied by serving the community. The desire to serve others and the feeling of volunteerism is ingrained within us and we do not leave any stone unturned when it comes to serving others. We are used to this habit now although there are many people who accuse us of being “bhatta khaane haru (per diem receivers).”

It was also evident that raising awareness on hypertension would bring additional benefits concerning other health problems in the community. One FCHV said:

This programme will provide us with the opportunity to inquire about other diseases, too. For example: If a pregnant woman comes to us to get her blood pressure examined, we can ask her about health issues other than those related to hypertension. So it will offer us an opportunity to take action in more ways.

### Theme 7: possible challenges for future involvement

FCHVs described one of the challenges of this programme to be access to medication and treatment after the detection of HBP. Some were also concerned about the harm they could cause by implying that someone has had HBP and thus at risk of a stroke. Some believed passing on bad news could harm their image as FCHVs. One said:

If people cannot handle the fact that they have high blood pressure and they have to buy medication to keep it under control, it can damage our images as FCHVs. We would be treated like the harbingers of bad news.

FCHVs also mentioned that financial hardship might make it difficult for people to comply with treatment. Issues such as returning to the doctor for a check-up are described by the FCHVs as:

I knew someone who was taking the medication on a regular basis, but he could not afford to go to the doctor, and he was not aware that the medications he was taking were not enough to curb his very high blood pressure.

Despite these challenges, FCHVs agreed that if they received the proper training to monitor blood pressure, awareness of the problem would be raised in their community and it would be possible for them also to advise people on how to prevent and assist in the management of hypertension. Even if some cannot afford the medicine it would still be possible for the FCHVs to guide people on how to control their blood pressure by a change in dietary and exercise habits.

## Discussion

This study is the first to report the overall literacy and motivation for control and prevention of hypertension among FCHVs. The current knowledge on the role of CHWs in preventing hypertension at community level is very limited as very few studies have explored the associations between knowledge of CHWs and their potential role in blood pressure management. In a study from Chile demonstrating the value of training CHWs in preventing NCDs, it was shown that limited training had not only increased knowledge of CHWs but also promoted their performance (13). An Iranian study demonstrated educational training in risk factors of cardiovascular disease had a sustained effect 6 years after training was initiated on modifying community lifestyle risk factors (14). Adams et al. (17) demonstrated health volunteers without a professional health worker background could be trained to make accurate blood pressure and anthropometric measurements. Reidpath et al. found that blood pressure readings made by non-health workers were not statistically different from those made by health workers (18). A study showed that in India, CHWs could be trained to calibrate blood pressure apparatus in the local setting, and that CHWs made similar BP measurements as their educated supervisors (19).

As mentioned earlier, it is a general problem for people in the communities where the FCHVs live and work to have access to equipment and training to provide blood pressure measurements. Lack of education and awareness as well as financial hardship and transportation issues present barriers to the measurement of blood pressure. We believe that blood pressure management till now has been a low priority in the communities of rural Nepal.

The FCHVs expressed willingness to take part in spreading community awareness on hypertension; in fact they stated that they have already been active in such activities. Although they seemed to hold some grudges about some community members' disbelief of their selfless service at times by linking their service with monetary incentives.

FCHVs constitute a link between different healthcare systems [the popular, the professional and the folk sectors as described by Kleinman (20)], and they exhibit an ability to navigate successfully in the grey zone between different healthcare practices (traditional and biomedical) (20). According to studies, FCHVs have a unique ability to provide successful healthcare to the members of their communities, because they share beliefs and general health-seeking behaviour with their patients/peers. The FCHVs are responsible for connecting their patients to the health post and educating the members of their communities about biomedical practices, but they possess a unique ability to provide healthcare that is concordant with the patient's life (21). They are able to engage in effective patient–provider communication, know and respect community norms and share habits with their patients, which allow them to empathise with those they serve (22). Thus, our study supports a future possibility of involving FCHVs in prevention and control of hypertension.

### Strength and limitations of the study

This study has some strengths and limitations. Its strengths that we adapted the guidelines used in earlier studies to ensure cultural acceptability. We believe that the primary author's familiarity with the local dialect and the local setting also helped ease the environment of discussion and thereby address the issue of cultural acceptability. During the process, an effort was made not to let the first author's own knowledge influence the response of the participants. We acknowledge that the small sample drawn from a single location limits the generalisability of the findings.

## Conclusions

FCHVs possess some knowledge about diagnosis, risk factors, and consequences of hypertension. It was a general belief that hypertension and its risk factors needed to be addressed. All FCHVs demonstrated a willingness to contribute to prevention, control, and management, regardless of the provision of incentives. However, there is a need for an intervention to train FCHVs on raising awareness of hypertension and its risk factors. Training would furthermore provide a point of contact for communities for hypertension-related cardiovascular disease or prevention and for seeking advice on treatment. There is currently no access to such a point of contact in the local communities.
